# Specialist LINK and primary care network clinical pathways - a new approach to patient referral: a cross-sectional survey of awareness, utilization and usability among family physicians in Calgary

**DOI:** 10.1186/s12875-020-01159-0

**Published:** 2020-05-12

**Authors:** Mubashir Arain, Mahnoush Rostami, Mariama Zaami, Valerie Kiss, Richard Ward

**Affiliations:** Health Systems Evaluation & Evidence, Innovation and Research Management, Research Innovation and Analytics, Alberta Health Services, Southport Tower, Calgary, AB T2W 1S7 Canada

**Keywords:** Specialist *LINK*, Primary care, Specialist referral, Clinical pathways

## Abstract

**Background:**

Specialist *LINK* is a real-time, non-urgent telephone collaboration line designed to link family doctors and specialists. The purpose was to reduce wait times, improve efficiency and enhance the coordination of patient care through enhanced communication between primary and specialty care. The aim of this study was to determine the awareness and utilization of Specialist *LINK* and Primary Care Network (PCN) Clinical Pathways among family physicians.

**Methods:**

A family physician experience cross-sectional survey was conducted from March to May 2018 in Calgary and Area. The survey was designed to assess family physicians’ awareness and utilization of Specialist *LINK* and PCN Clinical Pathways. We also used a 1–10 scale for respondents to rate the utility of Specialist *LINK* (1 was least useful and 10 represented highly useful). To obtain a true representative sample, family physicians were selected through a random sampling method. We applied multiple approaches to ensure a high response rate: paper survey, telephone reminders, and an on-site survey for non-responders.

**Results:**

A total of 251 participants completed the survey of the 650 randomly selected family physicians (Response rate≈39%). Eighty-nine percent of the family physicians were aware of Specialist *LINK* [95% Confidence Interval (84–92%)]. The average rating was 8.1 (on a scale of 1–10) for the usefulness of Specialist *LINK*. We found that the odds of being aware of Specialist *LINK* were two times higher in female family physicians compared to male physicians. Also, those with less than 5 years of experience, the odds of being aware of Specialist *LINK* were around five times higher compared to those with 5 or more years of experience. Fifty-five percent of family physicians were aware of PCN Clinical Pathways (95% CI = 48–60%); of those, 82% were accessing and following PCN Clinical Pathways in their clinical practice. The average rating was 7.9 (on a scale of 1–10) for the usefulness of PCN Clinical Pathways.

**Conclusion:**

Most of the respondents in Calgary and area were aware of Specialist *LINK* and a large proportion of them were using it to access advice for their patients.

## Background

Waiting for specialist care is one of the leading barriers in health service delivery in Canada [1, 2]. Long wait times may cause many challenges for patients, such as increased pain and suffering, as well as mental distress. These challenges may consequently lead to potentially irreversible chronic illnesses or injuries, or even permanent disabilities [2, 3]. When a patient is referred to a specialist by a primary care provider, the delay in such appointments could result in concerns for patients and their families. One study reported that the total wait time^1^ between referral from a general practitioner and delivery of treatment by a specialist, averaged across all 12 specialties and 10 provinces surveyed, raised from 20.0 weeks in 2016 to 21.2 weeks in 2017 [1]. The study also reported that the average wait time to see a specialist after referral by a primary care provider has increased significantly over the past years from 3.7 weeks in 1993 to and 8.9 weeks in 2010. A recent report indicated that a patient had to wait 4.5 years to see a neurologist at Kingston General Hospital in Ontario [4].

To reduce specialist wait times and improve access to specialty care for patients without the need for a face-to-face visit, electronic consultation was introduced in Winchester, Ontario [3, 5, 6]. This e-consultation system gave primary care providers the opportunity to submit a patient-specific clinical question to a specialist using a standardized web-based form. The electronic consultation increased patient and care providers’ satisfaction, improved access to specialist care, and reduced wait times.

Alberta introduced Specialist *LINK* to provide timely access to specialists through a real-time, non-urgent telephone consultation process. Specialist *LINK* is currently available to family physicians in Calgary and Area. The standard for specialists is to return the doctor’s call within 1 hour. This allows family physicians to talk to specialists and seek advice for a patient’s care plan. The aim was to reduce unnecessary specialist visits when a patient can be managed by a family physician through telephone discussion [7].

### Development of Specialist LINK and clinical pathways

Primary care networks (PCNs) are stand-alone organizations jointly owned by a group of family physicians practicing in Alberta. The main goal of PCNs is to increase access to primary care services and better coordination of primary health services with other healthcare services, such as hospitals and specialists. The telephone advice line (Specialist *LINK*) was developed through the Health Systems Support working group, comprised of representatives from Alberta Health Services (AHS) and PCNs, and specialists [8]. The first phone line was conducted in late 2014 with Gastrointestinal (GI) specialists. The feedback from both GI specialists and family doctors indicated that there were benefits of collaborative treatment plans. Since 2014, the partnerships have increased to include a number of other specialties: Chronic Pain Clinic, Congestive Heart Failure, Endocrinology, Gastroenterology, Hepatology, Nephrology, Neurology, Podiatric Surgery, Pediatrics, Psychiatry, Respirology, Rheumatology, and Vascular Surgery.

The PCNs in Calgary and Area also developed enhanced Clinical Pathways together with AHS and specialists. PCN Clinical Pathways are web-based tools to provide information on diagnosis and treatment options for family physicians [8]. These guidelines are designed to better equip family doctors with the knowledge and support they need to determine if a patient needs referral to a specialist or if that patient could be managed by a family physician. The pathways represent evidence-based best practice but do not override the individual responsibility of health care professionals to make decisions appropriate to their patients’ condition using their own clinical judgment. In several cases, these pathways help physicians limit the number of specialist referrals without compromising the quality of care. One example is Gastroenterology, where long waits to see specialist for common gastroenterology problems, particularly when patients do not report concerning symptoms (e.g. weight loss, rectal bleeding, anemia or iron deficiency). Five Gastroenterology Pathways were developed for common GI conditions to help family physician make correct decision regarding referral: irritable bowel syndrome, dyspepsia, gastroesophageal reflex disease, chronic constipation and Helicobacter Pylori infection. Preliminary results indicated that the non-urgent GI wait-list dropped from 2742 in January 2016 to 30 by February 2018 through the implementation of clinical pathways [8].

The primary focus of this study was to determine the awareness and utilization of Specialist *LINK* and PCN Clinical Pathways among family physicians in Calgary and Area.

## Methods

We conducted a cross-sectional survey [9, 10] from March to May 2018 in seven PCNs of Calgary, Alberta. We used three distribution methods to ensure a high response rate, a true representation of the target population, and to obtain valid results: paper survey through the mail, telephone survey, and onsite visits.

A minimum of a 235 physician sample was required to have statistically significant findings at a 10% margin of error, with 50% response distribution. A 50% response distribution was used to get the maximum sample. We used random sampling to obtain a representative sample of family physicians in Calgary. Each PCN provided the list of family physicians. We used random sampling and generated random numbers through Microsoft Excel. Physicians who moved out of the province or retired were replaced.

The survey was developed by the authors and sent to the Medical Director, Alberta Health Services and Executive Directors of Primary Care Networks for their feedback to test its face validity. The questionnaire consisted of awareness and utilization questions regarding Specialist *LINK* and PCN Clinical Pathways, along with some demographic questions. We also used a 1–10 scale for respondents to rate the utility of the Specialist *LINK* (1 represented not useful and 10 represented highly useful). To obtain a better response rate, most questions asked participants to choose the best answer and only a few were open-ended questions. We also added an “Other” category for close-ended questions to capture those responses not listed in the provided options.

### Phase one - paper survey

We mailed a paper survey to the potential participants (*n* = 650 randomly selected). We provided them with two options to return the completed survey: use a pre-paid return envelope or fax the survey. Preliminary discussions with the key stakeholders revealed that fax is the most commonly used communication methods by physician offices.

We replaced 22 surveys which were mailed back and returned to us (1 retired; 6 specialists; 2 locum, 2 on maternity leave; 1 out of the country; 10 no longer practice at the clinic). We received a total of 159 completed surveys during this phase: 121 by mail and 36 by fax.

### Phase two - telephone survey

We conducted a telephone survey for those participants who did not respond to the mailed survey. At the beginning of this phase, we conducted a pilot telephone survey to test our telephone script. Our survey lab conducted 35 phone calls (five calls to each PCN). As a result, we modified the telephone script to improve the response rate during the telephone survey phase. Overall, our survey lab conducted 491 phone calls to either remind the physicians or resend the survey by fax to those who did not receive the survey by mail or misplaced it. During this phase, we replaced 25 participants (2 retired; 5 on leave; 3 specialists, 13 no longer practice at the clinic). As a result of the phone calls, we received a total of 48 surveys: 33 by fax, 12 by mail, and 3 were completed over the phone.

### Phase three - on-site survey

During the third phase, we conducted an on-site survey at physicians’ clinics. We visited 134 clinics during this phase. As a result of these visits, we completed 22 surveys on-site and received 24 completed surveys by fax.

#### Data analysis

Survey responses were analyzed through descriptive and inferential statistical analysis using SPSS version 19. T-test was used for comparing continuous data and the chi-square test was used for categorical data. It came up in preliminary analysis that some of the apparently significant differences could potentially be solely due to confounding factors. Therefore, logistic regression model was used to determine the effect of gender, urban/rural areas, and years of experience on the awareness and utilization of Specialist *LINK* and PCN Clinical Pathways.

Also, we coded open-ended responses and examined patterns in respondent characteristics. Findings were used to interpret, explain and provide context for the quantitative findings while pertinent examples were drawn out to provide explanation and depth to the findings.

This study was considered as a Quality Improvement project and did not require approval by an ethics review board. However, all data collection, management and storing procedures complied with the Health Information Act and the Freedom of Information and Privacy Act. All participants were provided with information on the project, how the data would be used and informed that their participation was voluntary. Informed consent was assumed on return of the survey. We also applied the Alberta Research Ethics Community Consensus Initiative (ARECCI) Tool on the Specialist *LINK* to assess the risk for the participants [11]. Alberta Innovates Health Solutions developed a four-step, web-based ARECCI Ethics Screening Tool to review the projects that involve obtaining health information from people and to provide practical decision-support assistance to project leaders and teams. The Specialist *LINK* project score indicated that the project involved minimal risk.

## Results

**Fig. 1:**
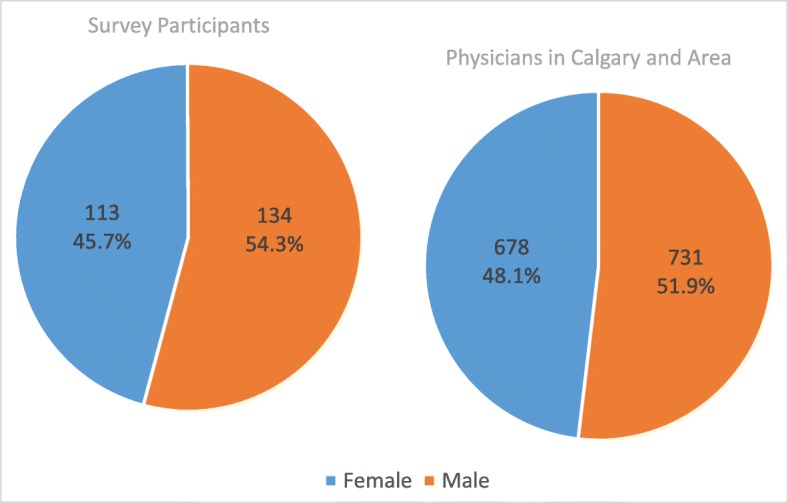
Gender Distribution of Survey Participants and Family Physicians in Calgary and Area

In total, 251 surveys were completed with a response rate of 39%. The majority of participants (64%) had more than 5 years of work experience as a family physician. Fifty-four percent of the participants were female. We compared the gender distribution of survey participants to all family physicians in Calgary to determine the representativeness of the survey sample (Fig. 1). No significant difference was found (54% vs. 52% male physicians; χ^2^ = 0.47; *p* value = 0.49).

**Fig. 2:**
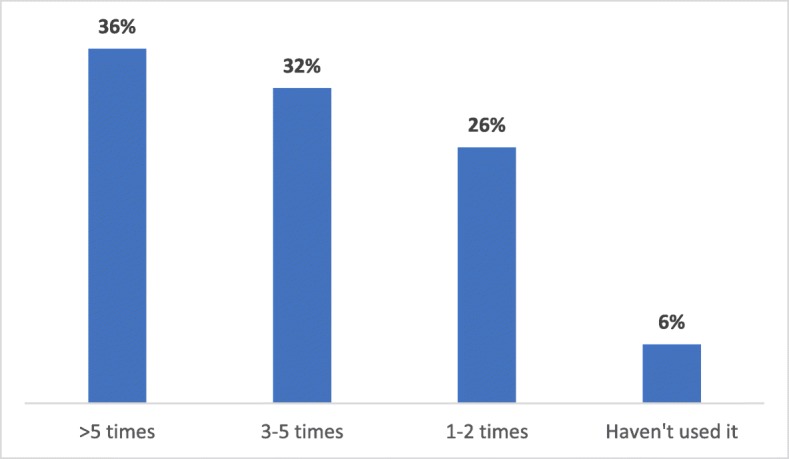
Frequency of Specialist *LINK* Utilization (*n* = 187)

**Fig. 3:**
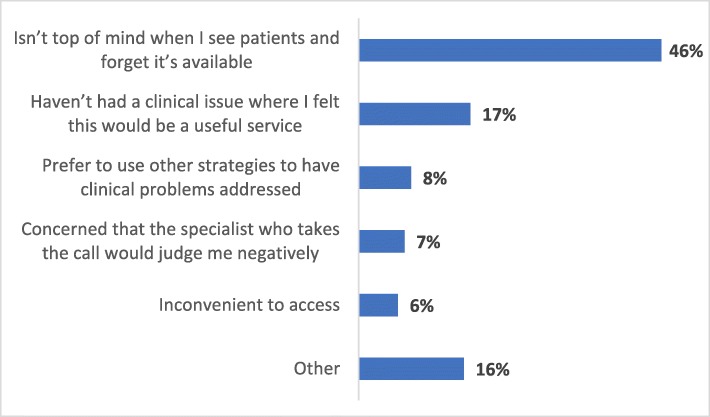
Reasons for Not Using Specialist *LINK* (*n* = 91)

Eighty-nine percent (215/242) of family physicians reported that they were aware of Specialist *LINK* (95% CI = 84–92%); of those, 72% had used it (158/219). Around 36% of the participants reported using Specialist *LINK* more than five times and only 6% reported being aware of it but never used it (Fig. 2). Forty-six percent of participants who did not use Specialist *LINK* said that the service was not at the top of their mind; around 17% did not have any relevant clinical issue, 7% perceived that specialists would judge them negatively, and 8% preferred to use other strategies to solve their clinical problems (Fig. 3).

Overall, 73% percent of the participants believed that Specialist LINK changed their patient management. Most of the family physicians rated the effectiveness of Specialist Link as an 8, 9, or 10 on a scale of 1–10 (mean score = 8.1; SD = 1.8; *n* = 187), where 1 indicates the least effective and 10 indicates the most effective.

### Awareness and utilization of PCN clinical Pathways

Around 55% (95% CI = 48–60%) of family physicians were aware of PCN Clinical Pathways (128/235). Those who were aware of PCN Clinical Pathways were asked to list any three PCN Clinical Pathways. Around 89% listed at least one correct PCN Clinical Pathway while 11% named Clinical Pathways that were not developed by PCNs.

**Fig. 4:**
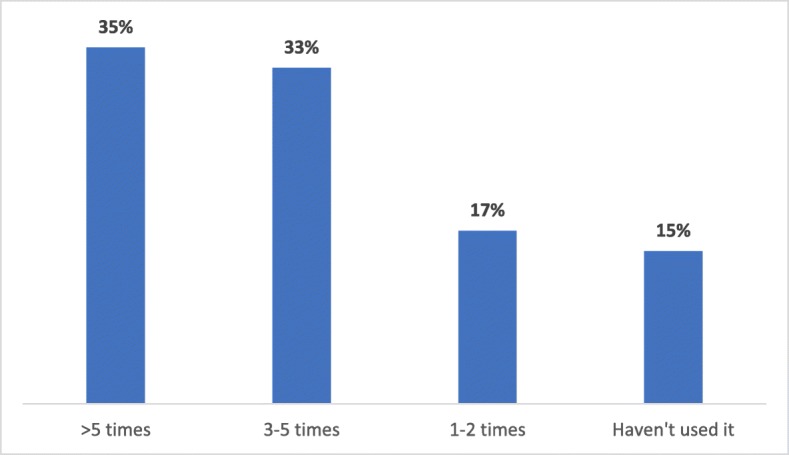
Frequency of PCN Clinical Pathways Utilization (*n* = 127)

**Fig. 5:**
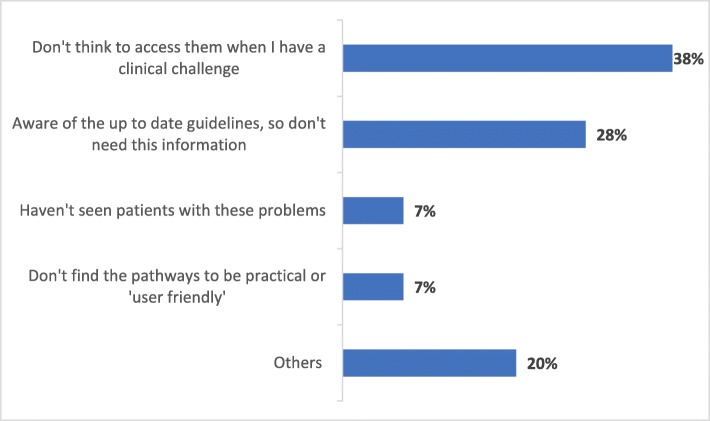
Reasons for Not Using PCN Clinical Pathways (*n* = 29)

Of those participants who were aware of PCN Clinical Pathways (sub-sample), around 82% reported that they accessed and followed PCN Clinical Pathways in their practice (114/139). Figure 4 shows that around 35% sub-sample participants had used PCN Clinical Pathways more than five times. Participants reported several reasons for being aware of PCN Clinical Pathways but not using them. Approximately 38% did not think of accessing them when they had a clinical challenge, 28% reported being aware of the up-to-date guidelines, and did not need the information, 7% had not seen patients with the problems listed on the clinical pathways, and 7% did not find the pathways to be practical or user-friendly (Fig. 5). Twenty percent of the participants had other reasoning, including having trouble finding PCN Clinical Pathways, not having time to use them, and one respondent wished to refer the patients to preferred specialists.

Questions were also asked regarding the effectiveness of PCN Clinical Pathways. Most of them (85%) rated the effectiveness of PCN Clinical Pathways as 7, 8, 9, or 10 on a scale of 1–10, where 1 indicates the least effective and 10 indicates the most effective. The average score was 7.8 (Mean = 7.8; SD = 1.6; *n* = 116).

### Differences in awareness and utilization of Specialist LINK and PCN Clinical Pathways

There was no difference in Specialist *LINK* awareness between rural and urban PCNs (χ^2^ = 0.12, *p* = 0.73); around 89% (*n* = 210) in urban compared to 91% (*n* = 32) of those in rural PCNs reported awareness of Specialist *LINK* (Table 1). The proportion of female physicians who were aware of Specialist *LINK* was significantly greater (93%) than the proportion of male physicians (84%) (χ^2^ = 5.19, *p*< 0.05) (Table 2).

**Table 1:** The comparison of Specialist LINK awareness and utilization between Physicians in Urban and Rural PCNs

	Urban PCNs n (%)	Rural PCNs n (%)	Chi-square Statistics	*P*-value
Awareness of Specialist *LINK*			0.12	0.73
Yes	186 (88.6%)	29 (90.6%)		
No	24 (11.4%)	3 (9.4%)		
Used Specialist *LINK*			0.66	0.42
Yes	136 (71.2%)	22 (78.6%)		
No	55 (28.8%)	6 (21.4%)		
Number of times accessed			0.82	0.37
Haven’t used it	11 (7.3%)	0 (0.0%)		
1–2 times	39 (25.8%)	7 (30.4%)		
3–5 times	49 (32.5%)	6 (26.1%)		
> 5 times	52 (34.4%)	10 (43.5%)		
Improvement in patient management			0.01	0.95
Yes	127 (73.0%)	21 (72.4%)		
No	47 (27.0%)	8 (27.6%)		
Effectiveness of Specialist *LINK* Mean (SD)	8.07 (1.82)	8.26 (1.57)	0.18^a^	0.68
	*n* = 164	*n* = 23		
Awareness of Clinical Pathways			3.63	0.06
Yes	117 (56.8%)	11 (37.9%)		
No	89 (43.2%)	18 (62.1%)		
Accessed PCN Clinical Pathways			0.14	0.71
Yes	102 (81.6%)	12 (85.7%)		
No	23 (18.4%)	2 (14.3%)		
Number of times used			0.54	0.46
Haven’t used it	17 (14.9%)	2 (15.4%)		
1–2 times	21 (18.4%)	1 (7.7%)		
3–5 times	38 (33.3%)	4 (30.8%)		
> 5 times	38 (33.3%)	6 (46.2%)		
Change in practice			2.11	0.15
Yes	90 (81.1%)	9 (64.3%)		
No	21 (18.9%)	5 (35.7%)		
Effectiveness of PCN Clinical Pathways Mean (SD)	7.96 (1.55)	7.55 (2.06)	0.21^a^	0.65
	*n* = 104	*n* = 11		

^a^T-test was used for comparison

*SD* Standard Deviation

**Table 2:** Comparing the awareness and utilization of Specialist LINK between Male and Female Physicians

	Male Physicians n (%)	Female Physicians n (%)	Chi-square Statistics	*P*-value
Awareness of Specialist *LINK*				
Yes	92 (83.6%)	120 (93.0%)	5.19	0.02^*^
No	18 (16.4%)	9 (7.0%)		
Used Specialist *LINK*			2.94	0.09
Yes	63 (66.3%)	93 (76.9%)		
No	32 (33.7%)	28 (23.1%)		
Number of times accessed			0.32	0.57
Haven’t used it	5 (7.2%)	6 (5.8%)		
1–2 times	18 (26.2%)	26 (25.2%)		
3–5 times	23 (33.3%)	32 (31.1%)		
> 5 times	23 (33.3%)	39 (37.9%)		
Improvement in patient management				
Yes	59 (67.8%)	88 (77.9%)	2.54	0.11
No	28 (32.2%)	25 (22.1%)		
Effectiveness of Specialist *LINK* Mean (SD)	7.60 (2.02)	8.48 (1.46)	3.62^a^	0.06
	*n* = 77	*n* = 106		
Awareness of Clinical Pathways				
Yes	49 (46.7%)	78 (61.5%)	5.03	0.03^*^
No	56 (53.3%)	49 (38.5%)		
Accessed Clinical Pathways			0.30	0.58
Yes	47 (79.7%)	65 (83.3%)		
No	12 (20.3%)	13 (16.7%)		
Number of times used			1.57	0.21
Haven’t used it	11 (21.2%)	8 (11.0%)		
1–2 times	8 (15.4%)	14 (19.2%)		
3–5 times	17 (32.7%)	23 (31.5%)		
> 5 times	16 (30.7%)	28 (38.3%)		
Change in practice			5.55	0.02^*^
Yes	37 (69.8%)	61 (87.1%)		
No	16 (30.2%)	9 (12.9%)		
Effectiveness of Clinical Pathways Mean (SD)	7.35 (1.85)	8.36 (1.24)	2.99^a^	0.07
	*n* = 46	*n* = 67		

^*^Significant differences (*p* < 0.05)

^a^T-test was used for comparison

*SD* Standard Deviation

Significant differences between years of work experience and awareness of Specialist *LINK* was found. Around 96% of those with less than 5 years of work experience compared to 85% of those with five or more years of work experience were aware of Specialist *LINK* (χ^2^ = 6.39, *p* < 0.05) (Table 3).

**Table 3:** The comparison of Specialist LINK awareness and utilization between those physicians with < 5 years of experience and those with 5 or more years of experience

	< 5 years n (%)	= > 5 years n (%)	Chi-square Statistics	*P*-value
Awareness of Specialist *LINK*			6.39	0.01^*^
Yes	77 (96.2%)	128 (85.3%)		
No	3 (3.8%)	22 (14.7%)		
Used Specialist *LINK*			0.05	0.83
Yes	58 (72.5%)	91 (71.1%)		
No	22 (27.5%)	37 (28.9%)		
Number of times accessed			0.01	0.95
Haven’t used it	2 (3.3%)	8 (7.8%)		
1–2 times	15 (24.6%)	25 (24.3%)		
3–5 times	25 (41.0%)	28 (27.2%)		
> 5 times	19 (31.1%)	42 (40.7%)		
Improvement in patient management			0.73	0.39
Yes	57 (77.0%)	85 (71.4%)		
No	17 (23.0%)	34 (28.6%)		
Effectiveness of Specialist *LINK* Mean (SD)	8.20 (1.46)	8.15 (1.84)	0.83^a^	0.36
	*n* = 65	*n* = 110		
Awareness of Clinical Pathways			0.00	0.97
Yes	43 (55.1%)	79 (54.9%)		
No	35 (44.9%)	65 (45.1%)		
Accessed Clinical Pathways			1.62	0.20
Yes	42 (87.5%)	66 (78.6%)		
No	6 (12.5%)	18 (21.4%)		
Number of times used			0.64	0.43
Haven’t used it	6 (12.8%)	12 (16.2%)		
1–2 times	12 (25.5%)	10 (13.5%)		
3–5 times	15 (31.9%)	22 (29.7%)		
> 5 times	14 (29.8%)	30 (40.6%)		
Change in practice			0.01	0.93
Yes	37 (80.4%)	59 (79.7%)		
No	9 (19.6%)	15 (20.3%)		
Effectiveness of Clinical Pathways Mean (SD)	7.83 (1.69)	8.06 (1.54)	0.47^a^	0.49
	*n* = 41	*n* = 65		

^*^Significant differences (*p* < 0.05)

^a^T-test was used for comparison

*SD* Standard Deviation

The first Logistic Regression Model (Table 4) shows the effect of demographic variables on the awareness of Specialist *LINK*. The model shows that the odds of female participants being aware of Specialist *LINK* was around two times higher than male participants (OR = 2.4; 95% CI = 1.1–5.9) after controlling for the effect of urban/rural clinic location and years of experience. The other statistically significant finding was around the years of experience; the odds of participants being aware of Specialist *LINK* was around five times higher (OR = 4.6; 95%CI = 1.3–16.3) for family physicians with less than 5 years of experience compared to those with 5 or more years of experience (after controlling for the effect of gender and urban/rural clinical setting).

**Table 4:** Logistic Regression of explanatory variables against the outcome “Aware of Specialist *LINK*” (*n* = 223)

	Adjusted Odds Ratios	95% Confidence Interval
Gender		(1.1–5.9)^a^
Male Physicians	1	
Female Physicians	2.4	
Clinic location		(0.3–4.6)
Urban	1	
Rural	1.2	
Work experience as family physician		(1.3–16.3)^a^
= > 5 yrs.	1	
< 5 yrs.	4.6	

^a^Statistically significant

Odds ratios were calculated after adjusting for other variables in the model

The second logistic regression model (Table 5) for the awareness of PCN Clinical Pathways shows no statistically significant differences in relation to gender and years of experience. Those working in a rural setting were 50% less likely to report being aware of PCN Clinical Pathways compared to those in urban settings; however, the difference was not statistically significant.

**Table 5:** Logistic Regression of explanatory variables against the outcome “Aware of PCN Clinical Pathways” (*n* = 216)

	Adjusted Odds Ratios	95% Confidence Interval
Gende		(0.9–2.8)
Male Physicians	1	
Female Physicians	1.6	
Clinic location		(0.2–1.2)
Urban	1	
Rural	0.5	
Work experience as family physician		(0.6–1.7)
= > 5 yrs.	1	
< 5 yrs.	0.9	

Odds ratios were calculated after adjusting for other variables in the model

## Discussion

The study results indicate that a large majority of respondents were aware of Specialist *LINK* and were using the service. This indicates that once family physicians are aware of a new service that directly help them in improving patient care, they are comfortable with using it. In addition, family physicians were very satisfied with their experience of using Specialist *LINK* and PCN Clinical Pathways, which supports the continuation of the service in the future.

The high awareness and utilization of Specialist *LINK* services also show that family physicians appreciate enhanced communication with specialists. The quick response from specialists through Specialist *LINK* encourages family physicians to use the service. The close support from specialty care enhances family physicians’ ability to manage patients in their office. In many cases, family physicians might be able to deal with patient problems but refer patients to specialists to make sure patients are getting the right treatment. Due to quick communication with specialists, family physicians would be able to look after many cases that otherwise would have been referred to specialists [3, 12]. As identified by Barua [1], and Liddy et al. [3], waiting for specialist care has been one of the major barriers to health care access in Canada [13]. This issue is directly addressed by the successful implementation of Specialist *LINK* and PCN Clinical Pathways.

We identified differences in awareness by the years of work experience. New family physicians tend to explore more options to improve clinical practice [14, 15], resulting in higher awareness of new programs and guidelines. It would be worth exploring further in future studies whether new family physicians find more issues with the current specialist referrals process, thus more likely to explore new options of specialist referrals compared to experienced family physicians. It was also observed that female physicians were more likely to be aware of Specialist *LINK*. Research shows that female doctors are more likely to adhere to new clinical guidelines than male doctors [16, 17]. Family physicians in rural areas were slightly less likely to be aware of PCN Clinical Pathways. Currently, there is an unequal distribution and under servicing of rural areas coupled with a shortage of specialists, as only 2.4% practice in rural areas [18].

This study has a few limitations. First, the response rate was low (around 39%) and the non-responders could have a different level of knowledge and awareness regarding Specialist *LINK* compared to those who responded to the survey. The reason for the low response rate could be the lack of time among family physicians to complete the survey. Second, some of the contact information for family physicians was not up-to-date which resulted in failed delivery of the paper survey to a few potential participants; this means those physicians were not included in the sampling framework. Lastly, approximately 11% of Clinical Pathways named by participants were not developed by PCN; there is a possibility that those who did not correctly name the PCN Clinical Pathways were unaware of PCN Clinical Pathways and mixed them up with other pathways not developed by PCNs.

## Conclusion

Most of the survey respondents were aware of Specialist *LINK* and a large proportion of them were using it for referring their patients. Female physicians and those with less than 5 years of experience were more likely to be aware of Specialist Link. The high uptake of these services shows their potential to significantly impact and improved the physician-specialist referral process. The improve referral process would increase patient satisfaction and access to health care services.

Future studies should try and engage physicians who did not respond to this study and also capture the perspective of specialists and patients about Specialist *LINK.*

## Data Availability

The datasets generated and/or analyzed during the current study are not publicly available due organizational data sharing restrictions but are available from the corresponding author on reasonable request. The sample of survey can be shared with interested parties upon request.

## Abbreviations

PCN: Primary Care Network
GI: Gastrointestinal
CI: Confidence Interval
SD: Standard Deviation

## Footnotes

1 A wait time begins with the booking of a service, when the patient and the appropriate physician agree to a service and the patient is ready to receive it. The appropriate physician is one with the authority to determine the needed service. A wait time ends with the commencement of the service (Ontario Ministry of Health and Long Term Care, 2005).
